# Association between tooth brushing habits and incident disability in community-dwelling older adults: a prospective cohort study

**DOI:** 10.1265/ehpm.25-00398

**Published:** 2026-03-25

**Authors:** Kimiko Tomioka, Midori Shima, Keigo Saeki

**Affiliations:** Nara Prefectural Health Research Center, Nara Medical University, Kashihara, Nara, Japan

**Keywords:** Oral self-care, Tooth brushing, Interdental cleaning tool, Incident disability, Prospective cohort study, Community-dwelling older adults, Japan

## Abstract

**Background:**

Japan is experiencing a rapid demographic shift toward a super-aged society, making the extension of healthy life expectancy a national priority. Oral self-care, particularly tooth brushing habits, may play a critical role in preventing functional decline among older adults, yet evidence in this population remains limited. This study aimed to examine the association between specific tooth brushing behaviors—frequency, timing (especially before bedtime), and use of interdental cleaning tools—and incident disability among community-dwelling older adults in Japan.

**Methods:**

A three-year prospective cohort study was conducted in City A, Nara Prefecture, involving 7,785 older adults aged 65 and above who were free of disability at baseline. Tooth brushing habits were assessed via self-administered questionnaires, and incident disability was defined as new certification under Japan’s Long-Term Care Insurance system. Covariates included age, gender, socio-economic status, lifestyle habits, physical and mental health, and oral health indicators. Multiple logistic regression analyses were used to estimate adjusted odds ratio (AOR) and 95% confidence interval (CI) for incident disability.

**Results:**

During the follow-up period, 713 participants (9.2%) developed incident disability. After adjustment for covariates and mutual adjustment for other tooth brushing habits, non-daily brushing before bedtime and non-use of interdental cleaning tools were associated with higher odds of incident disability (AOR for non-daily vs daily brushing before bedtime = 1.55, 95% CI: 1.19–2.01; AOR for non-use vs use of interdental cleaning tools = 1.34, 95% CI: 1.11–1.62). These associations were particularly evident among individuals aged 75 and older.

**Conclusion:**

Daily brushing before bedtime and the use of interdental cleaning tools were independently associated with lower odds of incident disability among community-dwelling older adults. Nevertheless, causal inference is limited, and residual confounding cannot be ruled out.

**Supplementary information:**

The online version contains supplementary material available at https://doi.org/10.1265/ehpm.25-00398.

## Background

Japan is currently a super-aged society, with 29.1% of its population aged 65 or older as of 2023—a figure expected to rise to 38.7% by 2070 [1]. In response to this demographic shift, extending healthy life expectancy has emerged as a critical public health priority. Preventing the onset of long-term care needs is essential not only for reducing medical and caregiving expenditures but also for improving the quality of life (QOL) among older adults [2].

Extensive research has demonstrated that having fewer remaining teeth in old age is linked to increased risks of mortality [3], cognitive decline [4], and functional disability [5]. While most investigations into tooth brushing habits have concentrated on dental caries in children and periodontal diseases in adults [6, 7], there remains a notable lack of research focused on older populations. Yet, infrequent tooth brushing has been linked to a range of adverse health outcomes, including cardiovascular and metabolic disorders [8], various forms of cancer [9], and systemic conditions such as diabetes, respiratory infections, and cognitive impairment [10]. A recent systematic review and meta-analysis further revealed that oral health indicators—including the number of remaining teeth, denture usage, brushing habits, tongue pressure, and occlusal force—are associated with frailty in older adults [11]. Tooth brushing represents a fundamental and modifiable self-care behavior that individuals can perform independently, making it a practical target for preventive health interventions. Identifying specific brushing habits that are independently associated with prolonged healthy lifespan is essential for designing effective, evidence-based strategies to promote healthy aging.

This study seeks to determine which specific tooth brushing behaviors—such as frequency, timing, and the use of dental aids—serve as independent predictors of incident disability among community-dwelling older adults. The results are expected to inform the development of evidence-based public health strategies aimed at extending healthy life expectancy.

## Methods

### Study area and participants

**Fig. 1:**
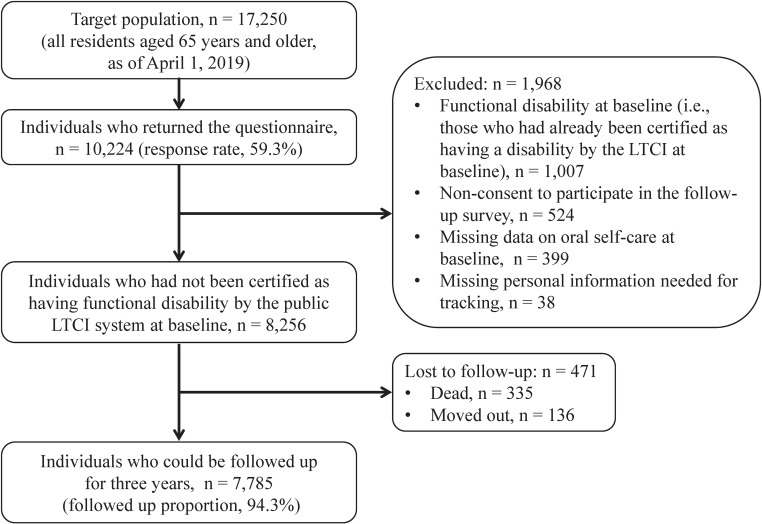
Flow chart of study participants. LTCI, Long-Term Care Insurance. Outcome not ascertained at follow-up (death/move-out) (n = 471)

The details of this cohort study are explained elsewhere [12]. Briefly, we conducted a baseline survey in October 2019 and a follow-up survey in October 2022. The study area was City A in Nara Prefecture. As of October 2019, the aging rate (the proportion of people aged 65 years or older in the total population) in this area was 23.0%, lower than the national average (28.4%). Potential study participants were all residents aged 65 years or older as of April 1, 2019 (n = 17,250). Exclusion criteria were those residing in a nursing home or undergoing long-term hospitalization. In October 2019, City A distributed self-administered questionnaires by mail to 17,250 individuals, of which 10,224 responded (response rate: 59.3%). A total of 8,256 individuals who were not certified as having functional disabilities under the public long-term care insurance (LTCI) system at baseline and who provided valid responses regarding oral self-care were eligible for follow-up. Incident disability was assessed only at the 3-year follow-up survey in October 2022; therefore, participants whose LTCI status at follow-up could not be ascertained (e.g., due to death or moving out of City A during follow-up) were excluded because we could not determine whether disability occurred before loss to follow-up. The final analytical sample comprised 7,785 individuals who were free of disability at baseline and whose disability status was available at follow-up (Fig. 1).

Comparing the basic attributes of respondents and non-respondents in the baseline survey (Additional Table 1), non-respondents were more likely to be female, in the youngest age group (65–69 years old), the oldest age group (85 years or older), and those with functional disability than respondents. When comparing the basic attributes between the final analyzed participants and each group of those excluded from the analysis (Additional Table 2), the group that did not agree to participate in the follow-up survey were mostly in the youngest age group (65–69 years old), and most of those with missing data on brushing teeth habits were 80 years or older. Those who were lost to follow-up (e.g., due to death or moving out during follow-up) were mostly male and aged 80 years or older.

### Assessment of outcome (incident disability)

Incident disability was assessed at the end of the 3-year follow-up period (October 2022) and defined as newly having LTCI certification at follow-up among participants without LTCI certification at baseline. Because the exact date of LTCI certification during follow-up was not available, incident disability was treated as a binary outcome at follow-up. LTCI certification is based on a nationally standardized assessment of functional capacity [13, 14]. Certification is determined by the Municipal Certification Committee, comprising experts in insurance, welfare, and medicine, following a comprehensive evaluation that includes a physical and mental health survey conducted by a certified investigator and a medical opinion provided by the attending physician. The committee assigns care needs using a seven-tier classification system: Support Levels 1–2 and Care Levels 1–5. Previous research has demonstrated a strong inverse correlation between LTCI certification levels and the Barthel Index (Spearman’s ρ = −0.86), supporting the validity of LTCI certification as an indicator of incident functional disability among older adults in Japan [15].

### Assessment of the independent variable (tooth brushing habits at baseline)

To assess older adults’ tooth brushing habits, we used three items based on previous research [6–11, 16–18]: the number of times brushing teeth per day, brushing teeth before bedtime, and the use of interdental cleaning tools. Participants were asked, “How many times a day do you brush your teeth?” and were categorized as 3+ times, 2 times, or 0–1 time per day. For brushing before bedtime, participants were asked, “Do you brush your teeth after dinner or before going to bed?” and indicated frequency (daily, sometimes, rarely, never); responses were dichotomized as daily versus not daily (sometimes/rarely/never). For interdental cleaning tools, participants were asked whether they used interdental brushes or dental floss in addition to toothbrushes (use versus no use). To assess multicollinearity among the three tooth brushing habits, variance inflation factors (VIFs) were calculated; all VIFs were ≤1.5.

### Covariates

Previous studies indicate that low socioeconomic position is associated with both higher risk of incident disability [19, 20] and lower prevalence of dental health behaviors [21]. Recent research has reported that lifestyle habits [22], physical and mental health [19], and oral health [23] are associated with maintaining healthy life expectancy in old age. Therefore, in this study, the following variables were included as covariates that may correlate with tooth brushing habits and incident disability: gender, age, socio-economic status (SES), lifestyle habits, physical and mental health, and oral health.

SES included family structure, perceived economic situation, and education. Lifestyle habits included dietary variety, exercise habits, and smoking history. Dietary variety was evaluated using the Dietary Variety Score (range 0–10) [24], and the lower tertile (0–3) was defined as low dietary variety. Physical health included chronic medical conditions and body mass index (BMI). Mental health was evaluated using the Kessler 6-item Psychological Distress Scale (K6) [25]. Psychological distress was defined as K6 ≥10 based on a recommended cut-off point [26]. For reference, Japan’s national health promotion plan, “Healthy Japan 21” (Phase 2), uses a threshold of K6 ≥10 [27]. Oral health consisted of three components: dental status, oral function, and regular dental visits. Dental status was classified into four groups based on the number of remaining teeth (≥20 vs ≤19) and denture use (use vs no use). Oral function was assessed with three questions [28]: chewing ability (Do you have difficulty chewing hard foods?), swallowing disorders (Do you choke on tea or soup?), and subjective dry mouth (Do you experience dry mouth?).

Drinking habits for lifestyle habits, and depression and cognitive functioning for mental health were not included as covariates because their variance inflation factors (VIF) exceeded 2.0. That is, all variables employed as covariates in this study had a VIF of 2.0 or less, confirming that there were no multicollinearity issues. Regarding the treatment of missing values in covariates, we followed statistical recommendations and used multiple imputation by chained equations [29]. Details of the covariates and multiple imputations are described in the supplementary data (Additional file 3), and information including the number of missing values according to gender is shown in Additional Table 4.

### Statistical analysis

Multiple logistic regression analysis was used to calculate adjusted odds ratio (AOR) and 95% confidence interval (CI) for incident disability. Baseline tooth brushing habits were the independent variable. In Model 1, all covariates were simultaneously included to estimate multivariable-adjusted AOR. In Model 2, to assess the independent association of each tooth brushing habit with incident disability, we additionally included all three tooth brushing variables in the same model for mutual adjustment. Because the exact date of LTCI certification during follow-up was not available and disability status at follow-up could not be ascertained for participants who died or moved out before the follow-up survey, time-to-event analyses and competing-risk analyses were not feasible; therefore, we estimated associations with 3-year cumulative incident disability using logistic regression among participants with disability status available at follow-up. Statistical analysis was performed using the IBM SPSS Statistics Ver. 27 for Windows (Armonk, NY, USA), and a significance level was set at 0.05 (two-tailed test).

### Sensitivity analysis for loss to follow-up (inverse probability weighting)

To evaluate potential selection bias arising from loss to follow-up (death or move-out), we conducted an inverse probability weighting (IPW) analysis among participants eligible for follow-up (n = 8,256). To estimate stabilized weights, we first fitted a logistic regression model for follow-up ascertainment, in which the dependent variable was whether disability status at follow-up was available (yes/no). Explanatory variables were the three baseline tooth brushing habits (number of times brushing teeth per day, brushing teeth before bedtime, and use of interdental cleaning tools) and the same covariates as in Model 1: gender, age, family structure, perceived economic situation, education, dietary variety, exercise habits, smoking history, chronic medical conditions, body mass index, psychological distress, dental status, chewing ability, swallowing disorders, subjective dry mouth, and regular dental visits. Stabilized weights were computed as the marginal probability of follow-up divided by each participant’s predicted probability. We then refitted the Model 2 logistic regression using these weights and the same set of covariates among participants with disability status available at follow-up (n = 7,785).

## Results

Among the 7,785 participants included in the analysis, the mean age (standard deviation) was 74.1 (5.9) years and the proportion of men was 45.8%. At the 3-year follow-up assessment (October 2022), 713 older adults newly received LTCI certification (the 3-year cumulative incidence of disability: 9.2%). The cumulative incidence was 7.7% (275/3,565) in men, 10.4% (438/4,220) in women, 2.9% (127/4,380) among those aged 65 to 74 years, and 17.2% (586/3,405) among those aged 75 years or older. Regarding baseline tooth brushing habits (Additional Table 5), significantly more men than women brushed their teeth less than twice daily (31.6% of men, 10.7% of women), did not brush their teeth every day before bedtime (24.3% of men, 9.8% of women), and did not use interdental cleaning tools (50.2% of men, 36.5% of women) (chi-squared test, *P* < 0.001 for all items). By age, people aged 75 and older had a lower frequency of tooth brushing and use of interdental cleaning tools than those aged 65 to 74, but there was no difference in brushing before bedtime. In addition, people aged 75 and older were more likely to wear dentures than those aged 65 to 74 (Additional Table 5) (38.6% for those aged 65 to 74 and 58.8% for those aged 75 and older; chi-squared test, *P* < 0.001). Regarding participant characteristics according to tooth brushing habits (Table 1), compared with those with favorable habits, those with unfavorable habits were significantly more likely to have a poor perceived economic situation, a low level of education, a low dietary variety, no exercise habits, current smokers, chronic illnesses, fewer than 20 remaining teeth, dentures, or poor chewing ability, and significantly less likely to visit the dentist regularly (chi-squared test, *P* < 0.001).

**Table 1:** Baseline characteristics of study participants by tooth brushing habits

	**By number of times brushing teeth ** **per day**				**By brushing teeth before bedtime**			**Interdental cleaning tools**		
		
	**3+ times** **(n = 2,013)**	**2 times** **(n = 4,195)**	**0–1 time** **(n = 1,577)**	***P*-value**	**Daily** **(n = 6,507)**	**Not daily** **(n = 1,278)**	***P*-value**	**Use** **(n = 4,454)**	**No use** **(n = 3,331)**	***P*-value**
**Socio-demographics & lifestyle habits & physical and mental health**										
Gender: men	36.9%	40.5%	71.3%	<0.001	41.5%	67.7%	<0.001	39.9%	53.7%	<0.001
Age: 75 years and older	45.5%	41.5%	47.3%	<0.001	44.0%	42.5%	0.339	40.5%	48.0%	<0.001
Family structure: living alone	13.3%	12.6%	11.9%	0.446	12.5%	13.4%	0.385	12.9%	12.3%	0.447
Perceived economic situation: poor	21.0%	23.6%	28.9%	<0.001	22.7%	30.6%	<0.001	21.7%	27.2%	<0.001
Education (years of schooling): <10	16.7%	18.8%	21.6%	0.001	17.9%	23.4%	<0.001	16.5%	21.9%	<0.001
Dietary variety: low	23.4%	34.2%	50.3%	<0.001	30.8%	54.2%	<0.001	29.1%	42.1%	<0.001
Exercise habits: absent	40.4%	45.6%	54.5%	<0.001	44.2%	55.6%	<0.001	40.9%	52.9%	<0.001
Smoking history: current smokers	4.5%	6.7%	16.4%	<0.001	6.1%	18.5%	<0.001	5.9%	11.1%	<0.001
Chronic medical conditions: present	53.9%	58.1%	64.4%	<0.001	57.1%	64.6%	<0.001	56.3%	61.0%	<0.001
Those with psychological distress	9.3%	9.2%	11.1%	0.080	9.3%	11.0%	0.085	8.6%	10.9%	<0.001
**Oral health (dental status & oral function & regular dental visits)**										
Number of remaining teeth: <20 teeth	48.8%	49.5%	60.6%	<0.001	50.2%	58.5%	<0.001	43.3%	62.5%	<0.001
Use of dentures: present	48.3%	45.2%	52.3%	<0.001	47.9%	45.1%	0.066	42.1%	54.5%	<0.001
Chewing ability: poor	19.3%	17.0%	27.5%	<0.001	18.2%	27.2%	<0.001	13.8%	27.6%	<0.001
Swallowing disorders: present	24.8%	24.3%	26.1%	0.381	24.7%	25.4%	0.626	25.4%	23.8%	0.107
Subjective dry mouth: present	28.5%	26.4%	30.0%	0.014	27.2%	29.8%	0.059	27.5%	27.9%	0.721
Regular dental visits: present	69.5%	57.7%	37.7%	<0.001	60.7%	36.5%	<0.001	72.0%	36.3%	<0.001

*P* values were based on the chi-squared test. Estimates for variables with missing values were obtained using multiple imputation.

**Fig. 2:**
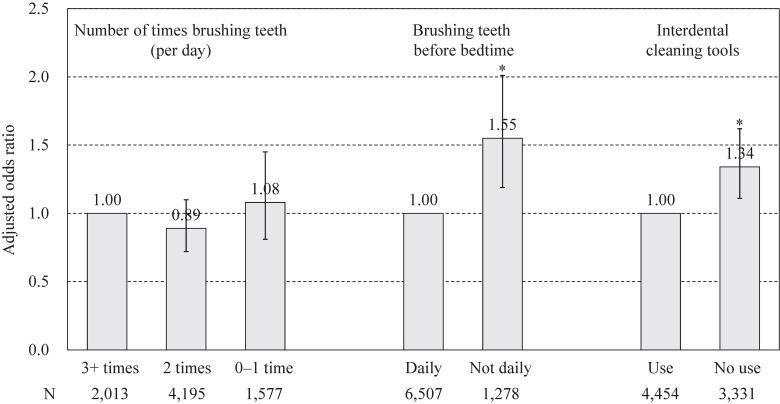
Adjusted odds ratio of tooth brushing habits at baseline for incident disability. Error bars display 95% confidence intervals. *P < 0.05. Adjusted for gender, age, family structure, economic status, education, dietary variety, exercise habits, smoking habit, medical history, BMI, mental status, dental status, chewing ability, swallowing disorders, subjective dry mouth, regular dental visits, the number of times brushing teeth, daily brushing teeth before bedtime, and the use of interdental cleaning tools.

Regarding the association of tooth brushing habits at baseline with incident disability, among all participants, significant associations were observed for all items in Model 1, which was adjusted for covariates (Additional Table 6). The AOR (95% CI) for brushing teeth 0–1 time per day compared with 3 or more times per day was 1.42 (1.10–1.83), the AOR (95% CI) for non-daily brushing teeth before bedtime versus daily brushing teeth before bedtime was 1.72 (1.38–2.14), and the AOR (95% CI) for non-use of interdental cleaning tools relative to use was 1.38 (1.15–1.67). In Model 2, which included mutual adjustment for other tooth brushing habits in addition to the covariates, the association for tooth brushing frequency was attenuated and no longer statistically significant, whereas significant associations remained for non-daily brushing before bedtime and non-use of interdental cleaning tools (AOR = 1.55, 95% CI = 1.19–2.01; and AOR = 1.34, 95% CI = 1.11–1.62, respectively) (Fig. 2). These significant associations were found in both men and women, but after stratified analysis by age, significant associations were only observed in those aged 75 and older, but not in those aged 65 to 74 (Table 2).

**Table 2:** Associations of tooth brushing habits at baseline with incident disability by gender or age

			**N**	**Cumulative** **Incidence** **rate^a^**	**Model 1**	**Model 2**	**N**	**Cumulative** **Incidence** **rate^a^**	**Model 1**	**Model 2**
			
					**Covariates adjusted**	**Mutually adjusted**			**Covariates adjusted**	**Mutually adjusted**
					**AOR^b^ (95% CI)**	**AOR^c^ (95% CI)**			**AOR^b^ (95% CI)**	**AOR^c^ (95% CI)**
**By gender**			**Men (n = 3,565)**				**Women (n = 4,220)**			
	
	Number of times brushing teeth (per day)	3+ times	743	6.5	1.00	1.00	1,270	10.3	1.00	1.00
		2 times	1,697	6.7	1.19 (0.81–1.75)	1.13 (0.77–1.66)	2,498	9.2	0.84 (0.65–1.08)	0.81 (0.62–1.04)^†^
		0–1 time	1,125	10.1	1.66 (1.11–2.47)*	1.25 (0.80–1.97)	452	17.3	1.36 (0.95–1.94)^†^	1.03 (0.68–1.55)

	Brushing teeth before bedtime	Daily	2,700	6.7	1.00	1.00	3,807	9.7	1.00	1.00
		Not daily	865	10.8	1.70 (1.25–2.28)**	1.50 (1.05–2.14)*	413	16.5	1.81 (1.29–2.52)**	1.64 (1.11–2.41)*

	Interdental cleaning tools	Use	1,775	5.0	1.00	1.00	2,679	8.4	1.00	1.00
		No use	1,790	10.4	1.55 (1.14–2.10)*	1.47 (1.08–2.00)*	1,541	13.8	1.31 (1.03–1.68)*	1.29 (1.01–1.65)*

**By age**			**Aged 65–74 (n = 4,380)**				**Aged 75 and older (n = 3,405)**			
	
	Number of times brushing teeth (per day)	3+ times	1,097	3.3	1.00	1.00	916	15.6	1.00	1.00
		2 times	2,452	2.2	0.64 (0.41–1.001)^†^	0.61 (0.39–0.96)*	1,743	16.6	1.02 (0.80–1.29)	0.97 (0.76–1.23)
		0–1 time	831	4.6	1.12 (0.66–1.92)	0.84 (0.44–1.58)	746	20.6	1.42 (1.06–1.90)*	1.08 (0.78–1.51)

	Brushing teeth before bedtime	Daily	3,645	2.5	1.00	1.00	2,862	16.1	1.00	1.00
		Not daily	735	4.9	1.67 (1.07–2.58)*	1.55 (0.90–2.66)	543	23.0	1.68 (1.30–2.18)**	1.55 (1.14–2.10)*

	Interdental cleaning tools	Use	2,649	2.5	1.00	1.00	1,805	13.8	1.00	1.00
		No use	1,731	3.6	1.26 (0.84–1.88)	1.23 (0.82–1.86)	1,600	21.1	1.44 (1.16–1.78)**	1.40 (1.12–1.73)*

AOR, adjusted odds ratio; CI, confidence interval. **P < 0.001, *P < 0.05, ^†^*P* < 0.10.

^a^The cumulative incidence rate of disability during the 3-year follow-up. ^b^Adjusted for covariates. ^c^Mutually adjusted for the three tooth brushing items in addition to covariates. Covariates included gender, age, family structure, economic status, education, dietary variety, exercise habits, smoking history, chronic medical conditions, BMI, psychological distress, dental status, chewing ability, swallowing disorders, subjective dry mouth, and regular dental visits.

In IPW-weighted analyses accounting for loss to follow-up, the associations of non-daily brushing before bedtime and non-use of interdental cleaning tools with incident disability were materially unchanged from the mutually adjusted primary models (Model 2), supporting the robustness of our findings (Additional Table 6): the IPW-weighted AORs were 1.56 (95% CI: 1.21–2.01) for non-daily brushing before bedtime and 1.35 (95% CI: 1.12–1.63) for non-use of interdental cleaning tools.

## Discussion

Using a three-year prospective cohort of community-dwelling older adults, we observed that non-daily brushing before bedtime and non-use of interdental cleaning tools were associated with higher odds of incident disability, even after adjustment for a wide range of sociodemographic factors, lifestyle habits, physical and mental health, and oral health indicators. However, because this was an observational study, the findings should be interpreted as associations rather than as evidence of a direct causal effect.

One possible explanation is that brushing before bedtime reduces the oral bacterial load overnight when salivary secretion decreases [30]. In addition, interdental brushes and dental floss can remove plaque between teeth and help prevent periodontal inflammation [31, 32]. Periodontitis may contribute to systemic inflammation and has been linked to cardiovascular disease and cognitive decline/dementia [33, 34]. Metabolic disorders such as diabetes are also associated with loss of disability-free survival [35]. Because these potential mediating factors were not directly assessed in our study, the observed associations may reflect an overall (total) association including indirect pathways, rather than a direct effect of tooth brushing habits on disability.

Another important consideration is residual confounding. Tooth brushing habits may function as proxy indicators of broader health-related behaviors and social factors. This study also showed that individuals with good tooth brushing habits had higher socio-economic status, a healthy lifestyle, fewer chronic medical conditions, better dental status, better chewing ability, and regular dental visits (Table 1). Although our models included socio-economic factors, lifestyle habits, physical and mental health, and oral health factors, unmeasured or imperfectly measured factors may remain, and therefore causal interpretation should be cautious.

Age-specific analyses showed that the association between tooth brushing habits and incident disability was apparent mainly among participants aged 75 and older. This may reflect increased systemic vulnerability in advanced age, making oral hygiene behaviors more relevant to maintaining functional health [36]. In addition, denture use is more prevalent among older age groups (Additional Table 5), and denture hygiene practices may also play an important role; however, denture hygiene behaviors were not specifically assessed in our study. These points underscore the need for age-tailored oral health interventions, particularly for those aged 75 and above [36, 37].

Regarding previous research on oral hygiene habits among community-dwelling older people, a 13-year follow-up of 14,206 Japanese adults aged 65 and older examined how well oral self-care practices (brushing twice a day or more, using dentures, and regular dental checkups) could compensate for the loss of healthy lifespan due to tooth loss, and reported that even with fewer teeth, oral self-care can extend healthy lifespan [38]. This study suggests that promoting oral self-care is effective in extending healthy lifespan, but it only evaluated brushing habits based on frequency. Another study that followed 21,881 individuals aged 75 and over who underwent dental checkups for an average of 3.5 years reported that those with good oral hygiene were less likely to develop functional disability compared to those with poor oral hygiene [39]. This study suggests that good oral hygiene contributes to a longer healthy lifespan in older people aged 75 and over. However, it only evaluated factors such as dental plaque adhesion and the cleanliness of dentures, and did not assess actual oral hygiene practices, including brushing habits.

Regarding tooth brushing frequency, a large Japanese cohort study involving 71,449 participants with a median follow-up of 5.2 years examined its association with cancer risk [9]. This previous study showed that brushing once or twice a day was associated with a lower cancer risk than brushing after every meal, suggesting that brushing after every meal may not be the most effective approach. In our study, tooth brushing frequency was significant in Model 1 but was attenuated in Model 2 after mutual adjustment for other tooth brushing habits. This attenuation likely reflects overlapping behaviors (e.g., individuals who brush more frequently may also be more likely to brush before bedtime), rather than clear evidence of no effect. Overall, our findings suggest the importance of not only frequency but also timing and quality of tooth brushing.

However, this study has several limitations. First, tooth brushing habits were self-reported, which may introduce reporting bias. Second, incident disability was defined using LTCI certification, which is based on a standardized assessment process, but it may not fully reflect actual changes in functional ability. For example, individuals who need assistance may not apply for or use LTCI services if informal care is available, potentially leading to outcome misclassification. Third, the baseline response rate was 59.3%, and participants who did not consent to the follow-up survey, had missing exposure data, or whose LTCI status at follow-up could not be ascertained (e.g., due to death or moving out during follow-up) were excluded from the analysis. These exclusions may have introduced selection/survivor bias. Moreover, because the exact date of LTCI certification during follow-up was unavailable and disability status could not be determined after loss to follow-up, we were unable to treat death or moving out as censoring events, and time-to-event analyses (including competing-risk approaches) were not feasible; therefore, the direction of any resulting bias is uncertain. However, in sensitivity analyses using IPW to address loss to follow-up, estimates closely matched those from the mutually adjusted primary models, suggesting that differential follow-up alone is unlikely to account for the observed associations. Fourth, because this study was conducted in a single municipality, there may be regional variation in LTCI certification, which may limit generalizability to other regions. Finally, as this was an observational study, causal inference is limited, residual confounding may remain, and indirect (mediated) pathways cannot be ruled out. Individuals who engage in oral self-care may also have healthier behaviors and social conditions that were not fully captured (e.g., diet, physical activity, daily routines/sleep-wake patterns, and social participation), and some oral hygiene practices (e.g., denture cleaning) were not assessed. Future studies using objective oral hygiene measures, information on denture hygiene and broader health behaviors, and analytic approaches that address censoring/competing risks are warranted.

## Conclusion

This large-scale prospective cohort study showed that brushing before bedtime and using interdental cleaning tools were independently associated with lower odds of incident disability among community-dwelling older adults, particularly among those aged 75 and older. Further studies are warranted to clarify potential indirect pathways and to address residual confounding.

## Abbreviations

AOR: adjusted odds ratio
BMI: body mass index
CI: confidence interval
IPW: inverse probability weighting
K6: Kessler 6-item Psychological Distress Scale
LTCI: Long-Term Care Insurance
SES: socio-economic status
VIF: variance inflation factor

## Supplementary information

**Additional file 1:** **Additional** **Table 1.** Basic attributes of respondents and non-respondents to the baseline survey. **Additional** **Table 2.** Comparison of basic attributes between final analyzed participants and each excluded group. **Additional file 3.** Detailed explanations of covariates and multiple imputations. **Additional Table 4.** Baseline characteristics of study participants by gender: before multiple imputation was performed. **Additional Table 5.** Baseline characteristics of study participants by gender and age. **Additional Table 6.** Associations of tooth brushing habits at baseline with incident disability among all participants (n = 7,785).

## Declarations

### Availability of data and materials

No data and materials are available.
