# Supplementary material for: Association between tooth brushing habits and incident disability in community-dwelling older adults: a prospective cohort study
Source: Environ Health Prev Med. 2026 Mar 25;31:22. doi: 10.1265/ehpm.25-00398 (PMC13057865; doi:10.1265/ehpm.25-00398)
Supplement: Supplementary file 1 — Additional file 1: Additional Table 1. Basic attributes of respondents and non-respondents to the baseline survey. Additional Table 2. Comparison of basic attributes between final analyzed participants and each excluded group. Additional file 3. Detailed explanations of covariates and multiple imputations. Additional Table 4. Baseline characteristics of study participants by gender: before multiple imputation was performed. Additional Table 5. Baseline characteristics of study participants by gender and age. Additional Table 6. Associations of tooth brushing habits at baseline with incident disability among all participants (n = 7,785). [file ehpm-31-022-s001.docx]

**Supplementary files**

**Additional Table 1**. Basic attributes of respondents and non-respondents to the baseline survey

| Basic attributes | | Respondents | Non-respondents | *P*-value^a^ |
| --- | --- | --- | --- | --- |
|  |  | (n = 10,224) | (n = 7,026) |  |
|  |  | n (%) | n (%) |  |
| Gender | |  |  |  |
|  | Men | 4,688 (45.9) | 3,034 (43.2) | 0.001 |
|  | Women | 5,536 (54.1) | 3,992 (56.8) |  |
| Age (years) | |  |  |  |
|  | 65–69 | 2,344 (22.9) | 1,785 (25.4) | <0.001 |
|  | 70–74 | 2,886 (28.2) | 1,869 (26.6) |  |
|  | 75–79 | 2,479 (24.2) | 1,472 (21.0) |  |
|  | 80–84 | 1,474 (14.4) | 951 (13.5) |  |
|  | ≥85 | 1,041 (10.2) | 949 (13.5) |  |
| Functional disability at baseline survey | | | |  |
|  | Absent | 9,217 (90.2) | 5,802 (82.6) | <0.001 |
|  | Present | 1,007 (9.8) | 1,224 (17.4) |  |

^a^ Chi-squared test.

**Additional Table 2**. Comparison of basic attributes between final analyzed participants and each excluded group

| Basic attributes | | Final analyzed  participants | Non-consent to  the follow-up survey | Missing data on  brushing teeth habits | Those who were  lost to follow-up |  | Comparison between two groups  based on chi-square test | | |
| --- | --- | --- | --- | --- | --- | --- | --- | --- | --- |
|  |  | (n = 7,785) | (n = 524) | (n = 399) | (n = 471) |  | Final analyzed participants vs. | | |
|  |  | n (%) | n (%) | n (%) | n (%) |  | Non-consent | Missing data | Lost to follow-up |
| Gender | |  |  |  |  |  |  |  |  |
|  | Men | 3,565 (45.8) | 248 (47.3) | 200 (50.1) | 288 (61.1) |  | *P* = 0.495 | *P* = 0.090 | *P* < 0.001 |
|  | Women | 4,220 (54.2) | 276 (52.7) | 199 (49.9) | 183 (38.9) |  |  |  |  |
| Age (years) | |  |  |  |  |  |  |  |  |
|  | 65–69 | 1,951 (25.1) | 155 (29.6) | 61 (15.3) | 69 (14.6) |  | *P* < 0.001 | *P* < 0.001 | *P* < 0.001 |
|  | 70–74 | 2,429 (31.2) | 185 (35.3) | 99 (24.8) | 97 (20.6) |  |  |  |  |
|  | 75–79 | 1,943 (25.0) | 121 (23.1) | 106 (26.6) | 121 (25.7) |  |  |  |  |
|  | 80–84 | 1,020 (13.1) | 43 (8.2) | 82 (20.6) | 97 (20.6) |  |  |  |  |
|  | ≥85 | 442 (5.7) | 20 (3.8) | 51 (12.8) | 87 (18.5) |  |  |  |  |

**Additional file 3.** Detailed explanations of covariates and multiple imputations

Covariates

Age was categorized as 65–74, 75–84, and ≥85 years. Family structure was categorized as living alone, living with only one’s spouse, living with a person other than a spouse, and living with three or more persons. Self-perceived economic status was categorized as rich (very rich/rich), fair, and poor (poor/very poor). Years of education was categorized as ≤9, 10–12, and ≥13 years. Dietary variety was dichotomized into low or not. Exercise habits was categorized as at least once a week, monthly or yearly, and almost never. Smoking history was categorized as never smoker, ex-smoker, and current smoker. Information on chronic medical conditions included hypertension, diabetes, stroke, heart disease, and cancer. The number of chronic medical conditions was categorized as none, one, and ≥two. Body mass index (kg/m^2^) was categorized as normal (18.5–24.9), thin (<18.5), and obese (≥25.0). Mental health was categorized into two groups based on the presence or absence of psychological distress. Dental status (the number of remaining teeth & the use of dentures) was categorized as ≥20 & no use, ≥20 & use, <20 & no use, and <20 & use. Chewing ability was dichotomized into good or poor. Swallowing disorders was dichotomized into present or absent. Subjective dry mouth was dichotomized into present or absent. Regular dental visits was dichotomized into present or absent.

Multiple imputations

Using logistic regression, we created five sets of imputation data and performed analyses on the complete pooled data set. The independent variable, outcome, gender, and all the covariate variables were entered into the imputation procedure. For this study, there were no missing data on gender, age, and regular dental visits. Family structure (1.0% missing), self-perceived economic status (1.3% missing), education (2.1% missing), dietary variety (0.3% missing), exercise habits (3.3% missing), smoking history (1.7% missing), chronic medical conditions (3.3% missing), body mass index (3.2% missing), psychological distress (4.0% missing), dental status (3.2% missing), chewing ability (0.2% missing), swallowing disorders (0.4% missing), and subjective dry mouth (0.6% missing) were imputed as ordinal variables.

**Additional Table 4.** Baseline characteristics of study participants by gender: before multiple imputation was performed.

|  |  |  | Men (n = 3,565) | Women (n = 4,220) | *P*-value^a^ |
| --- | --- | --- | --- | --- | --- |
|  |  |  | n (%) | n (%) |  |
| **Socio-demographics** | |  |  |  |  |
|  | Age (years) | 65–74 | 1,954 (54.8) | 2,426 (57.5) | 0.024 |
|  |  | 75–84 | 1,415 (39.7) | 1,548 (36.7) |  |
|  |  | ≥85 | 196 (5.5) | 246 (5.8) |  |
|  | Family structure | Living alone | 244 (6.8) | 727 (17.2) | <0.001 |
|  |  | Living with only  one’s spouse | 1,962 (55.0) | 1,800 (42.7) |  |
|  |  | Living with a person  other than a spouse | 93 (2.6) | 362 (8.6) |  |
|  |  | Living with three or  more persons | 1,216 (34.1) | 1,300 (30.8) |  |
|  |  | Missing | 50 (1.4) | 31 (0.7) |  |
|  | Self-perceived | Rich | 530 (14.9) | 640 (15.2) | 0.890 |
|  | economic status | Fair | 2,134 (59.9) | 2,535 (60.1) |  |
|  |  | Poor | 853 (23.9) | 995 (23.6) |  |
|  |  | Missing | 48 (1.3) | 50 (1.2) |  |
|  | Years of education | ≤9 | 619 (17.4) | 803 (19.0) | <0.001 |
|  | (years) | 10–12 | 1,530 (42.9) | 2,267 (53.7) |  |
|  |  | ≥13 | 1,339 (37.6) | 1,063 (25.2) |  |
|  |  | Missing | 77 (2.2) | 87 (2.1) |  |
| **Lifestyle habits** | |  |  |  |  |
|  | Low dietary variety | Absent | 2,060 (57.8) | 3,010 (71.3) | <0.001 |
|  |  | Present | 1,494 (41.9) | 1,198 (28.4) |  |
|  |  | Missing | 11 (0.3) | 12 (0.3) |  |
|  | Exercise habits | At least once a week | 1,347 (37.8) | 1,661 (39.4) | <0.001 |
|  |  | Monthly or yearly | 618 (17.3) | 455 (10.8) |  |
|  |  | Almost never | 1,497 (42.0) | 1,952 (46.3) |  |
|  |  | Missing | 103 (2.9) | 152 (3.6) |  |
|  | Smoking history | Never smoker | 926 (26.0) | 3,747 (88.8) | <0.001 |
|  |  | Ex-smoker | 2,060 (57.8) | 298 (7.1) |  |
|  |  | Current smoker | 504 (14.1) | 117 (2.8) |  |
|  |  | Missing | 75 (2.1) | 58 (1.4) |  |

**Additional Table 4**. Continued.

|  |  |  | Men (n = 3,565) | Women (n = 4,220) | *P*-value^a^ |
| --- | --- | --- | --- | --- | --- |
|  |  |  | n (%) | n (%) |  |
| **Physical and mental health** | | |  |  |  |
|  | Number of | None | 1,223 (34.3) | 1,925 (45.6) | <0.001 |
|  | chronic medical | One | 1,539 (43.2) | 1,674 (39.7) |  |
|  | conditions | ≥ two | 703 (19.7) | 461 (10.9) |  |
|  |  | Missing | 100 (2.8) | 160 (3.8) |  |
|  | Body mass index | Normal:18.5–24.9 | 2,500 (70.1) | 2,882 (68.3) | <0.001 |
|  | (kg/m^2^) | Thin: <18.5 | 140 (3.9) | 362 (8.6) |  |
|  |  | Obese: ≥25.0 | 817 (22.9) | 836 (19.8) |  |
|  |  | Missing | 108 (3.0) | 140 (3.3) |  |
|  | Psychological distress (K6)^†^ | Not distressed | 3,160 (88.6) | 3,605 (85.4) | <0.001 |
|  |  | Distressed | 261 (7.3) | 444 (10.5) |  |
|  |  | Missing | 144 (4.0) | 171 (4.1) |  |
| **Oral health** | |  |  |  |  |
|  | Dental status | ≥20 & no use | 1,235 (34.6) | 1,658 (39.3) | <0.001 |
|  | (Number of remaining teeth & denture use) | ≥20 & use | 383 (10.7) | 377 (8.9) |  |
|  |  | <20 & no use | 474 (13.3) | 593 (14.1) |  |
|  |  | <20 & use | 1,389 (39.0) | 1,429 (33.9) |  |
|  |  | Missing | 84 (2.4) | 163 (3.9) |  |
|  | Chewing ability | Good | 2,805 (78.7) | 3,433 (81.4) | <0.001 |
|  |  | Poor | 760 (21.3) | 771 (18.3) |  |
|  |  | Missing | 0 (0.0) | 16 (0.4) |  |
|  | Swallowing disorders | Absent | 2,712 (76.1) | 3,125 (74.1) | 0.002 |
|  |  | Present | 848 (23.8) | 1,071 (25.4) |  |
|  |  | Missing | 5 (0.1) | 24 (0.6) |  |
|  | Subjective dry mouth | Absent | 2,580 (72.4) | 3,018 (71.5) | 0.715 |
|  |  | Present | 963 (27.0) | 1,176 (27.9) |  |
|  |  | Missing | 22 (0.6) | 26 (0.6) |  |
|  | Regular dental visits | Present | 1,892 (53.1) | 2,524 (59.8) | <0.001 |
|  |  | Absent | 1,673 (46.9) | 1,696 (40.2) |  |

^†^ Psychological distress was defined as scoring ≥10 on the K6 scale.

**Additional Table 5.** Baseline characteristics of study participants by gender and age

|  |  | By gender | | | | | |  | By age | | | | | |
| --- | --- | --- | --- | --- | --- | --- | --- | --- | --- | --- | --- | --- | --- | --- |
|  |  | Men (n = 3,565) | |  | Women (n = 4,220) | | *P*-value |  | Aged 65–74 (n = 4,380) | |  | Aged ≥75 (n = 3,405) | | *P*-value |
|  |  | N | % |  | N | % |  |  | N | % |  | N | % |  |
| **Socio-demographics & lifestyle habits & physical and mental health** | | | | | | |  |  |  |  |  |  |  |  |
| Family structure: living alone | | 250 | 7.0% |  | 733 | 17.4% | <0.001 |  | 465 | 10.6% |  | 520 | 15.3% | <0.001 |
| Perceived economic situation: poor | | 864 | 24.2% |  | 1,006 | 23.8% | 0.686 |  | 1,089 | 24.9% |  | 781 | 22.9% | 0.048 |
| Education (years of schooling): <10 | | 634 | 17.8% |  | 830 | 19.7% | 0.038 |  | 501 | 11.4% |  | 964 | 28.3% | <0.001 |
| Dietary variety: low | | 1,498 | 42.0% |  | 1,201 | 28.5% | <0.001 |  | 1,621 | 37.0% |  | 1,078 | 31.7% | <0.001 |
| Exercise habits: absent | | 1,548 | 43.4% |  | 2,035 | 48.2% | <0.001 |  | 1,932 | 44.1% |  | 1,651 | 48.5% | <0.001 |
| Smoking history: current smokers | | 513 | 14.4% |  | 118 | 2.8% | <0.001 |  | 434 | 9.9% |  | 197 | 5.8% | <0.001 |
| Chronic medical conditions: present | | 2,311 | 64.8% |  | 2,227 | 52.8% | <0.001 |  | 2,302 | 52.6% |  | 2,236 | 65.7% | <0.001 |
| Those with psychological distress | | 280 | 7.9% |  | 468 | 11.1% | <0.001 |  | 332 | 7.6% |  | 416 | 12.2% | <0.001 |
| **Oral health (dental status & oral function & regular dental visits)** | | | | | | |  |  |  |  |  |  |  |  |
| Number of remaining teeth: <20 teeth | | 1,906 | 53.5% |  | 2,107 | 49.9% | 0.002 |  | 1,979 | 45.2% |  | 2,034 | 59.7% | <0.001 |
| Use of dentures: present | | 1,813 | 50.9% |  | 1,881 | 44.6% | <0.001 |  | 1,690 | 38.6% |  | 2,003 | 58.8% | <0.001 |
| Chewing ability: poor | | 760 | 21.3% |  | 775 | 18.4% | 0.001 |  | 668 | 15.3% |  | 867 | 25.5% | <0.001 |
| Swallowing disorders: present | | 849 | 23.8% |  | 1,078 | 25.5% | 0.085 |  | 1,024 | 23.4% |  | 904 | 26.5% | 0.001 |
| Subjective dry mouth: present | | 969 | 27.2% |  | 1,184 | 28.1% | 0.400 |  | 1,109 | 25.3% |  | 1,044 | 30.7% | <0.001 |
| Regular dental visits: present | | 1,892 | 53.1% |  | 2,524 | 59.8% | <0.001 |  | 2,490 | 56.8% |  | 1,926 | 56.6% | 0.818 |
| **Tooth brushing habits** | |  |  |  |  |  |  |  |  |  |  |  |  |  |
| No of times  brushing teeth | 3+ times per day | 743 | 20.8% |  | 1,270 | 30.1% | <0.001 |  | 1,097 | 25.0% |  | 916 | 26.9% | <0.001 |
|  | 2 times per day | 1,697 | 47.6% |  | 2,498 | 59.2% |  |  | 2,452 | 56.0% |  | 1,743 | 51.2% |  |
|  | 0–1 time per day | 1,125 | 31.6% |  | 452 | 10.7% |  |  | 831 | 19.0% |  | 746 | 21.9% |  |
| Brushing teeth before bedtime: not daily | | 865 | 24.3% |  | 413 | 9.8% | <0.001 |  | 735 | 16.8% |  | 543 | 15.9% | 0.339 |
| Interdental cleaning tools: no use | | 1,790 | 50.2% |  | 1,541 | 36.5% | <0.001 |  | 1,731 | 39.5% |  | 1,600 | 47.0% | <0.001 |

*P*-values were based on the chi-squared test. Estimates for variables with missing values were obtained using multiple imputation.

**Additional Table 6.** Associations of tooth brushing habits at baseline with incident disability among all participants (n = 7,785)

|  |  | N | Cumulative  Incidence  rate^a^ | Model 1 | Model 2 |  | IPW-weighted analyses |
| --- | --- | --- | --- | --- | --- | --- | --- |
|  |  |  |  | Covariates adjusted | Mutually adjusted |  | Mutually adjusted |
|  |  |  |  | AOR^b^ (95% CI) | AOR^c^ (95% CI) |  | AOR^c^ (95% CI) |
| Number of times  brushing teeth  (per day) | 3+ times | 2,013 | 8.9 | 1.00 | 1.00 |  | 1.00 |
|  | 2 times | 4,195 | 8.2 | 0.94 (0.76–1.15) | 0.89 (0.72–1.10) |  | 0.90 (0.73–1.11) |
|  | 0–1 time | 1,577 | 12.2 | 1.42 (1.10–1.83)^*^ | 1.08 (0.81–1.45) |  | 1.09 (0.81–1.45) |
| Brushing teeth  before bedtime | Daily | 6,507 | 8.5 | 1.00 | 1.00 |  | 1.00 |
|  | Not daily | 1,278 | 12.6 | 1.72 (1.38–2.14)^**^ | 1.55 (1.19–2.01)^*^ |  | 1.56 (1.21–2.01)^**^ |
| Interdental  cleaning tools | Use | 4,454 | 7.0 | 1.00 | 1.00 |  | 1.00 |
|  | No use | 3,331 | 12.0 | 1.38 (1.15–1.67)^**^ | 1.34 (1.11–1.62)^*^ |  | 1.35 (1.12–1.63)^*^ |

AOR, adjusted odds ratio; CI, confidence interval; IPW, inverse probability weighting. ^**^*P* < 0.001, ^*^*P* < 0.05.

^a^ The cumulative incidence rate of disability during the 3-year follow-up. ^b^ Adjusted for covariates. ^c^ Mutually adjusted for the three tooth brushing items in addition to covariates. Covariates included gender, age, family structure, economic status, education, dietary variety, exercise habits, smoking history, chronic medical conditions, body mass index, psychological distress, dental status, chewing ability, swallowing disorders, subjective dry mouth, and regular dental visits. IPW analyses used stabilized weights based on the predicted probability of remaining under follow-up. The follow-up ascertainment model used to derive the stabilized IPW included the three baseline tooth brushing habits and the same covariates as in Model 1.
